# UNCERTAINTY IN HOME HEALTH CARE FOR OLDER VETERANS: VA PERSPECTIVES ON AGENCY SELECTION AND CONTRACTING

**DOI:** 10.1093/geroni/igae098.3914

**Published:** 2024-12-31

**Authors:** Chelsea Leonard, Kate Magid, Lexus Ujano- De Motta, Marguerite Daus, Cari Levy, Christine Jones

**Affiliations:** VA Eastern Colorado Healthcare System, Aurora, Colorado, United States; Rocky Mountain Regional VA, Aurora, Colorado, United States; VA Eastern Colorado Healthcare System, aurora, Colorado, United States; Department of Veterans Affairs, Aurora, Colorado, United States; CU Anschutz, Aurora, Colorado, United States; VA Eastern Colorado Healthcare System, Aurora, Colorado, United States

## Abstract

Older Veterans discharged from the hospital with skilled home health care (HHC) have their HHC delivered by community home health agencies (HHA) and financed primarily through the Department of Veteran’s Affairs (VA) or Medicare. We sought to understand perspectives of VA personnel on the process of HHC agency selection and financing for Veterans referred for HHC. We used a sequential mixed methods study design and conducted in-person and virtual site visits at three VA Medical Centers (VAMCs) in the highest decile of VA-paid HHC services (3 in-person) and three VAMCs in the highest decile of Medicare-paid HHC services (1 in-person, 2 virtual). We observed processes related to HHC referrals and conducted interviews with key informants. Interviews were recorded, transcribed, and analyzed using inductive-deductive content analysis to identify themes related to referral processes, agency selection, and financing. We conducted 29 interviews with 39 personnel at 6 VAMCs. We identified 4 themes related to uncertainty around agency and payor selection: 1) VA roles are siloed and next steps are a “black box;” 2) VA personnel are uncertain how HHAs are selected for local networks; 3) Local VAMCs develop strategies to determine HHA quality and reputation; and 4) Veterans’ role in selecting HHA or financing is unclear. Understanding points of uncertainty has implications for VA’s ability to provide high-quality HHC for older Veterans. Our findings suggest that providing accessible information on HHA quality and clear guidance around Veterans’ role in HHA and payor selection may streamline the process of providing HHC to Veterans.

